# Supplementary figures and images for: Epidemiological evidence relating risk factors to chronic obstructive pulmonary disease in China: A systematic review and meta-analysis
Source: PLoS One. 2021 Dec 28;16(12):e0261692. doi: 10.1371/journal.pone.0261692 (PMC8714110; doi:10.1371/journal.pone.0261692)

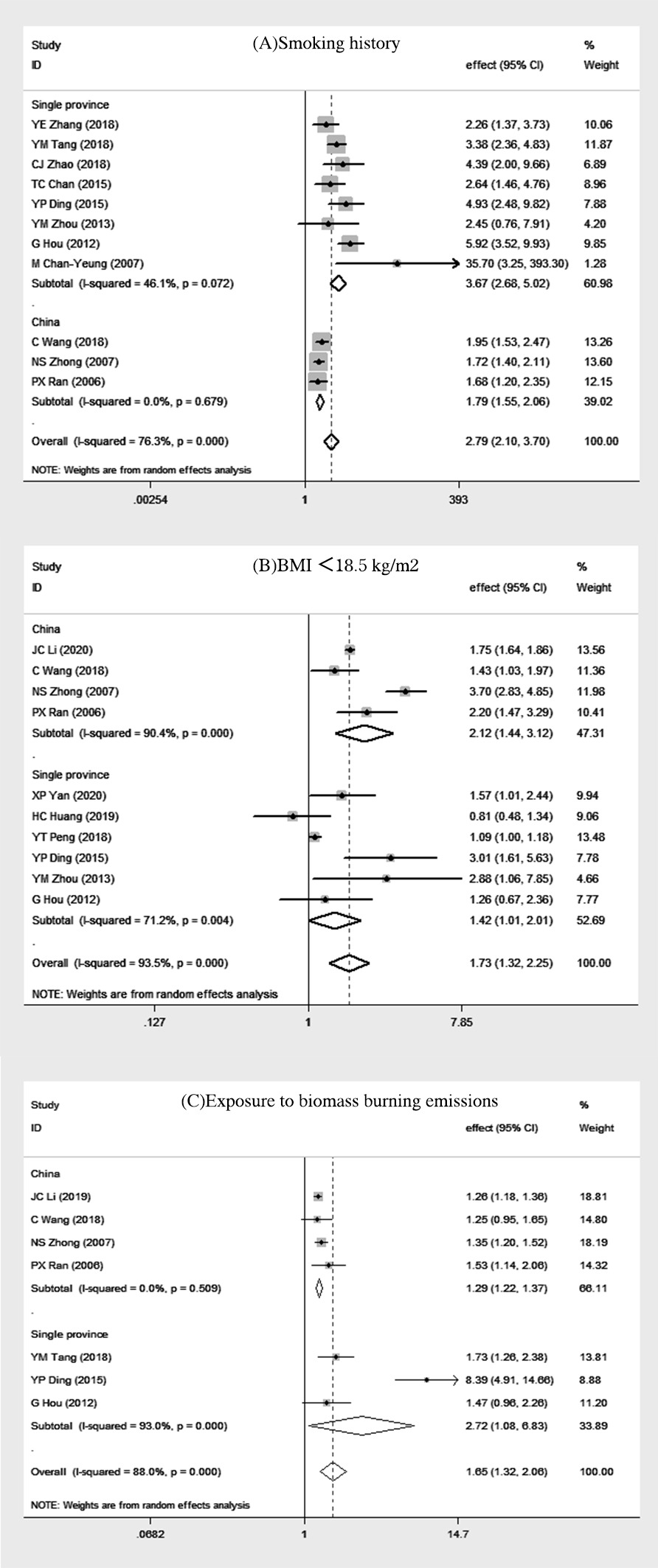

Supplement: S1 Fig — (TIF) [file pone.0261692.s002.tif]
