# Supplementary material for: Epidemiological evidence relating risk factors to chronic obstructive pulmonary disease in China: A systematic review and meta-analysis
Source: PLoS One. 2021 Dec 28;16(12):e0261692. doi: 10.1371/journal.pone.0261692 (PMC8714110; doi:10.1371/journal.pone.0261692)
Supplement: S1 Table — (DOCX) [file pone.0261692.s003.docx]

**S1 Table. Search strategy in PubMed**

#1 COPD[Title/Abstract]

#2 Chronic Obstructive Pulmonary Disease [Title/Abstract]

#3 “COPD”[MeSH]

#4 #1 OR #2 OR #3

#5 chronic bronchitis[Title/Abstract]

#6 emphysema[Title/Abstract]

#7 bronchial asthma[Title/Abstract]

#8 asthma[Title/Abstract]

#9 #5 OR #6 OR #7 OR #8

#10 #4 OR #9

#11 risk[tiab]

#12 risks[tiab]

#13 “Risk Factors”[MeSH]

#14 Exposure[tiab]

#15 cause[tiab]

#16 predict*[tiab]

#17 factor[tiab]

#18 factors[tiab]

#19 #11 OR #12 OR #13 OR #14 OR #15 OR #16 OR #17 OR #18

#20 #10 AND #19

#21 case-control[tiab]

#22 cohort*[tiab]

#23 cross sectional[tiab]

#24 population-based[ti]

#25 surveillance[tiab]

#26 “Case-Control Studies”[MeSH]

#27 “Cohort Studies”[MeSH]

#28 “Cross Sectional Studies”[MeSH]

#29 #21 OR #22 OR #23 OR #24 OR #25 OR #26 OR #27 OR #28

#30 #19 AND #29

#32 #20 AND #29
