# Supplementary material for: Epidemiological evidence relating risk factors to chronic obstructive pulmonary disease in China: A systematic review and meta-analysis
Source: PLoS One. 2021 Dec 28;16(12):e0261692. doi: 10.1371/journal.pone.0261692 (PMC8714110; doi:10.1371/journal.pone.0261692)
Supplement: S2 Table — (DOCX) [file pone.0261692.s004.docx]

**S2 Table. Publication bias associated with potential risk factors for COPD.**

| **Risk factors** | **Number of studies** | ***P*** |
| --- | --- | --- |
| PM2.5 exposure | 3 | 0.296 |
| Smoking history | 12 | 1.000 |
| Passive smoking history | 4 | 1.000 |
| Drinking history | 2 | 1.000 |
| Gender | 7 | 1.000 |
| BMI<18.5 kg/m^2^ | 10 | 0.592 |
| BMI≥28 kg/m^2^ | 8 | 0.902 |
| Biomass burning exposure | 7 | 0.035 |
| Childhood respiratory infections | 4 | 1.000 |
| Residence | 5 | 0.462 |
| Family history of respiratory diseases | 5 | 0.806 |

^a^BMI, Body mass index
